# A role for CETP TaqIB polymorphism in determining susceptibility to atrial fibrillation: a nested case control study

**DOI:** 10.1186/1471-2350-7-39

**Published:** 2006-04-19

**Authors:** Folkert W Asselbergs, Jason H Moore, Maarten P van den Berg, Eric B Rimm, Rudolf A de Boer, Robin P Dullaart, Gerjan Navis, Wiek H van Gilst

**Affiliations:** 1 Department of Cardiology, University Medical Center Groningen, Groningen, The Netherlands; 2 Department of Nutrition, Harvard School of Public Health, Boston, MA, USA; 3 Computational Genetics Laboratory, Department of Genetics, Department of Community and Family Medicine, Dartmouth Medical School, Lebanon, NH, USA; 4 Department of Epidemiology, Harvard School of Public Health, Boston, MA, USA; 5 Channing Laboratory, Department of Medicine, Brigham & Women's Hospital, and Harvard Medical School, Boston, MA, USA; 6 Department of Endocrinology, University Medical Center Groningen, Groningen, The Netherlands; 7 Department of Nephrology, University Medical Center Groningen, Groningen, The Netherlands; 8 Department of Clinical Pharmacology, University Medical Center Groningen, Groningen, The Netherlands

## Abstract

**Background:**

Studies investigating the genetic and environmental characteristics of atrial fibrillation (AF) may provide new insights in the complex development of AF. We aimed to investigate the association between several environmental factors and loci of candidate genes, which might be related to the presence of AF.

**Methods:**

A nested case-control study within the PREVEND cohort was conducted. Standard 12 lead electrocardiograms were recorded and AF was defined according to Minnesota codes. For every case, an age and gender matched control was selected from the same population (n = 194). In addition to logistic regression analyses, the multifactor-dimensionality reduction (MDR) method and interaction entropy graphs were used for the evaluation of gene-gene and gene-environment interactions. Polymorphisms in genes from the Renin-angiotensin, Bradykinin and CETP systems were included.

**Results:**

Subjects with AF had a higher prevalence of electrocardiographic left ventricular hypertrophy, ischemic heart disease, hypertension, renal dysfunction, elevated levels of C-reactive protein (CRP) and increased urinary albumin excretion as compared to controls. The polymorphisms of the Renin-angiotensin system and Bradykinin gene did not show a significant association with AF (p > 0.05). The TaqIB polymorphism of the CETP gene was significantly associated with the presence of AF (p < 0.05). Using the MDR method, the best genotype-phenotype models included the combination of micro- or macroalbuminuria and CETP TaqIB polymorphism, CRP >3 mg/L and CETP TaqIB polymorphism, renal dysfunction and the CETP TaqIB polymorphism, and ischemic heart disease and CETP TaqIB polymorphism (1000 fold permutation testing, P < 0.05). Interaction entropy graph showed that the combination of albuminuria and CETP TaqIB polymorphism removed the most entropy.

**Conclusion:**

CETP TaqIB polymorphism is significantly associated with the presence of AF in the context of micro- or macroalbuminuria, elevated C-reactive protein, renal dysfunction, and ischemic heart disease.

## Background

Atrial fibrillation (AF) is the most common sustained arrhythmia in clinical practice and is associated with an increase in total and cardiovascular mortality, as well as cardiovascular morbidity, including stroke and heart failure. [1]. It is therefore important to detect and treat AF at an early stage to prevent refractory AF and cardiovascular events in the future. Currently, our knowledge on the early pathophysiological changes in the atria is limited due to lacking understanding of molecular mechanisms. Studies in genetics of AF may provide some new insights in the etiology of AF. The familial form of AF is uncommon and AF is more often related to structural abnormalities. Therefore, analysis of AF as monogenic disease in family members with AF as primary electrical disease will tell us more about the etiology of AF, but cohort studies comparing cases to age and gender matched controls may explain the development of AF and identify genetic factors that predispose subjects to AF in combination with structural abnormalities [2]. Recently, activation of the renin-angiotensin system (RAS) and higher inflammatory state has been associated with AF in the future. [3,4]. Activation of RAS may cause the development of structural abnormalities partly by degrading bradykinin, which normally has a cardioprotective effect by diminishing the development of fibrosis. [5]. It was already known from studies in experimental AF that inhibition of the RAS prevents adverse atrial remodeling. [6]. Furthermore, genetic variations in the renin angiotensin system genes and inflammatory genes are associated with AF indicating the complexity of the development of AF [7,8]. Genetic variations in the cholesteryl ester transfer protein (CETP) gene are also interesting candidates to play an important role in cardiovascular disease. CETP enables the transfer of cholesteryl esters from high density lipoprotein (HDL) towards lipoproteins of lower density, thereby lowering HDL cholesterol. [9]. A high CETP concentration may be a determinant of increased cardiovascular risk in subjects with high triglycerides [10]. Variations in the CETP gene could be involved in the development of cardiovascular disease by increasing CETP activity and thereby reducing the cholesterol content of HDL relative to low density lipoprotein (LDL). Furthermore, a decreased CETP activity might prevent oxidation of LDL and decrease inflammation of the vascular wall through its effect on HDL [11]. Accumulating evidence suggests that CETP polymorphisms may be linked to coronary artery disease, as well as hypertension, which are both strongly linked to the incidence of AF. [12,13].

It is increasingly clear that the relationship between the genome and susceptibility to cardiovascular disease is complex [14]. As such, genetic analysis methods are needed that embrace, rather than ignore, this complexity [15]. In this case-control study, we investigate the association between several environmental factors and loci of candidate genes, which might be related to the presence of AF. In addition to the traditional univariate analyses, we explored the effects of gene-gene and gene-environment interactions on AF using the multifactor dimensionality reduction (MDR) method and creating an interaction entropy graph [16].

## Methods

### Subjects

The population analyzed in this study was obtained from the PREVEND study. [17]. The PREVEND (Prevention of Renal and Vascular Endstage Disease) study was designed to investigate the natural course of microalbuminuria and its relation with renal and cardiovascular disease in the general population. [17]. All inhabitants of the city of Groningen (Netherlands) between age 28–75 were asked to send in a morning urine sample and to fill in a short questionnaire on demographics and cardiovascular medical history. A total of 40,856 subjects responded. Subjects who were pregnant or using insulin were excluded. All subjects with a urinary albumin concentration ≥ 10 mg l^-1 ^(n = 7,768) and a randomly selected sample of subjects with a urinary albumin concentration <10 mg l^-1 ^(n = 3,395) were invited to perform two visits at an outpatient clinic. The screening program was completed by 8,592 subjects. From these, we selected all patients with AF on the electrocardiogram (n = 97). For every case, an age and gender matched control was selected from the PREVEND cohort. All participants gave written informed consent. The PREVEND study was approved by the local medical ethics committee and conducted in accordance with the guidelines of the declaration of Helsinki.

### Electrocardiography

Standard 12-lead electrocardiograms were recorded using the computer program MEANS (Modular ECG Analysis System) [18], and AF was defined according to Minnesota codes 8.3.1 and 8.3.3. Infarct patterns, suggestive of myocardial infarction, were defined by Minnesota codes 1.1 and 1.2. Major ischemia was defined by Minnesota codes 4.1, 4.2, 5.1, or 5.2. The presence of left ventricular hypertrophy (LVH) was identified using the Cornell voltage-duration product, which was calculated as follows: RaVL ± SV_3 _(with 6 mm added in women) × QRS duration. A threshold of 2440 mm·msec was used to identify LVH.

### Laboratory measurements

The urinary albumin excretion rate was measured as the mean of two 24-h urine collections, and microalbuminuria was defined as an albumin excretion rate between 30 and 300 mg per 24 h and macroalbuminuria > 300 mg per 24 h. Urinary albumin concentrations were determined by nephelometry with a threshold of 2.3 mg l^-1 ^and intra- and inter-assay coefficients of variation of less than 2.2% and 2.6%, respectively (Dade Behring Diagnostic, Marburg, Germany). High-sensitive C-reactive protein (CRP) was also determined by nephelometry with a threshold of 0.175 mg l^-1 ^and intra- and inter-assay coefficients of less than 4.4% and 5.7%, respectively (BNII N, Dade Behring). CRP levels below the detection level were scored as 0.18 mg l^-1^. Plasma glucose, serum cholesterol, serum and urinary creatinine were determined by Kodak Ektachem dry chemistry (Eastman Kodak, Rochester, NY, USA). HDL-cholesterol was measured with a homogeneous method (direct HDL, no. 7D67, AEROSET System; Abbott Laboratories). Serum triglycerides were measured enzymatically. [9]. LDL-cholesterol is estimated from quantitative measurements of total and HDL-cholesterol and triglycerides using the empirical relationship of Friedewald et al. [19]. Urinary leukocyte and erythrocyte measurements were done by Nephur-test+leuco sticks (Boehringer Mannheim, Mannheim, Germany).

### Genotyping of the RAS, Bradykinin and CETP gene polymorphisms

The AGT A-6G (rs5051) single nucleotide polymorphisms was analyzed using PCR primers and dual labeled TaqMan (non-MGB) probes as described earlier. [20]. The AT1R A-1166C (rs5186), the BDKRB2 C-58T (rs1799722) and C-181T (rs1046248), and the CETP Taq1B (rs708272) and I-405V (rs5882) polymorphisms were analyzed using TaqMan-MGB probes and PCR primers, designed through the Assay-by-Design service (Applied Biosystems). The ACE I/D and the BDKRB2 exon 1 insertion/deletion polymorphisms were also analyzed by PCR amplification, one of the primers having a fluoresent label, and subsequent determination of the lengths of the PCR products on a capillary sequencer. Primers were picked with the aid of online Primer3 software [21]. After PCR cycling, samples of the above assays were pooled and separated on a MegaBACE 1000 sequencer. Fragments were analyzed with Genetic Profiler 2.0 software (Amersham Biosciences).

### Definition of risk factors

Hypertension was defined as a systolic blood pressure ≥ 140 mm Hg or a diastolic blood pressure ≥ 90 mm Hg or the use of antihypertensive medication. Hypercholesterolemia was defined as a total serum cholesterol level of ≥ 6.5 mmol/l or the use of lipid-lowering therapy. Diabetes was defined as a fasting plasma glucose level >7.0 mmol/l or a non-fasting plasma glucose level >11.1 mmol/l or the use of antidiabetic medication. Obesity was defined as a body mass index ≥ 30 kg/m^2^. Smoking was categorized as no smoking or current smoking (current or stopped <1 year ago). Creatinine clearance (CrCl) was calculated as the mean of two 24-h urine creatinine excretions divided by plasma creatinine. Creatinine clearance was adjusted for body surface area, BSA = 0.007184 × weight^0.425 ^× length^0.725^, by dividing CrCl by BSA. Moderate renal dysfunction was defined as CrCl <60 mL/min/1.73 m^2 ^or the presence of microalbuminuria. Presence of ischemic heart disease was defined as prior myocardial infarction with hospitalization reported by questionnaire and/or an infarct and/or major ischemia patterns on the electrocardiogram.

### Statistical analysis

Continuous data are reported as mean (standard deviation) or median (interquartile range) if the data was skewed. Categorical data are presented as per group percentages. Differences between subgroups were evaluated by Student's *t*-test for the normally distributed continuous variables, or by the Mann-Whitney test if data was skewed. Differences in genotype frequencies and other categorical data between cases and controls were compared with the χ^2 ^test or Fisher's exact test. Consistency of genotype frequencies with the Hardy-Weinberg equilibrium (HWE) was tested using a chi-squared goodness-of-fit test on a contingency table of observed versus expected genotype frequencies in cases and controls. The expected genotype frequencies were calculated using allele frequencies estimated from the control sample of the entire PREVEND cohort. When departure of HWE was detected in the control group, the Armitage test for trend in proportions was used which does not assume HWE [22]. Genotype-phenotype associations were examined with dominant, and recessive models using logistic regression. Odds ratios for the presence of AF on the electrocardiogram and their 95% Confidence intervals were calculated. All *p*-values are two-tailed. A p-value of <0.05 was considered statistically significant. All abovementioned calculations were performed with SPSS version 12.0.1 software (SPSS, Chicago, IL).

For the evaluation of gene-gene and gene-environment interactions, we used the MDR method [23-26]. This method includes a combined cross-validation/permutation-testing procedure that minimizes false-positive results that might otherwise result from multiple examinations of the data [27]. Cross-validation divides the data into a training set and a testing set. With 10-fold cross-validation, the data are divided into 10 equal parts, and the model is developed on 9/10 of the data (training set) and then tested on 1/10 of the remaining data (testing set). This is repeated for each possible 9/10 and 1/10 of the data, and the resulting 10 testing accuracies are averaged. In addition to the testing accuracy, we also report the cross-validation consistency (CVC) that is a measure of how many times out of 10 divisions of the data that MDR found the same best model. Models that are true-positives are likely to generalize to independent datasets and will have estimated testing accuracies of greater than 0.5. Permutation testing was performed to assess the probability of obtaining a testing accuracy as large or larger than observed in the original data given the null hypothesis of no association is true. This is carried out by randomizing the case-control labels 1000 times and repeating the MDR analysis on each randomized dataset. This process yields an empirical distribution of testing accuracies under the null hypothesis that is in turn used to calculate a p-value. We report here the significant two-factor models. The MDR analysis was carried out using version 0.5.1 of the open-source MDR software package that is freely available. [28].

The following cardiovascular risk factors predisposing to AF were included in the MDR analysis: hypertension, hypercholesterolemia, obesity, diabetes, current smoking, family history for cardiovascular disease, HDL-cholesterol < 1 mmol/l, alcohol intake > = 1 drink per day, CRP > 3 mg/L, presence of micro- or macroalbuminuria, renal dysfunction, electrocardiographic left ventricular hypertrophy and ischemic heart disease.

The following genotypes were included in the MDR analysis: ACE gene insertion/deletion (I/D) polymorphism, the -6GA polymorphism of the Angiotensin (AGT) gene, the A1166C polymorphism of the angiotensin II type 1 receptor gene (AT1R), Bradykinin B2 receptor gene -81CT, -58CT, 9bp insertion/deletion polymorphisms, and the CETP TaqIB, I405V polymorphisms.

An interaction graph using entropy (measurement of randomness) estimates as described by Jakulin and Bratko. [29] and Moore et al [16] will be created to confirm, visualize and interpret the results obtained by logistic regression analysis and MDR. Interaction graphs are compromised of a node for each attribute with pairwise connections between them. The percentage of entropy removed by each attribute is visualized for each node and the percentage of entropy removed for each pairwise product of attributes is visualized for each connection. Thus, the independent main effects of each factor can be compared to the interaction effect and whether interactions are additive or non-additive can be quickly determined. Positive entropy values indicate synergistic interaction and negative entropy values indicate redundancy. The interaction graphs are created using Orange machine learning software package, which is written in Python and provided for free as open-source [30].

## Results

The clinical characteristics of cases and controls are shown in Table 1. Subjects with AF had a higher prevalence of electrocardiographic LVH, ischemic heart disease, hypertension, renal dysfunction, and higher levels of CRP and urinary albumin excretion as compared to age and gender matched controls.

**Table 1:** Baseline characteristics of the nested case-control study divided by control group of subjects without atrial fibrillation (AF) and cases with AF. Data shown as percentages for categorical variables and mean ± standard deviation for continuous variables. HDL-cholesterol, Triglycerides, Creatinine, C-reactive protein, and urinary albumin excretion are expressed as median [interquartile range].

	**AF Controls (n = 97)**	**AF Cases (n = 97)**	**P-value**
Age, years	59 ± 1	60 ± 1	Matched
Male	56.7%	56.7%	Matched
Caucasian	91.8%	92.8%	0.79
Body Mass Index	27.1 ± 0.4	27.8 ± 0.4	0.24
Current smoking	28.9%	30.2%	0.84
Ischemic heart disease	15.8 %	38.0 %	0.001
Left ventricular hypertrophy	3.1%	11.5%	0.03
Systolic blood pressure	135 ± 2	137 ± 2	0.47
Diastolic blood pressure	76 ± 1	79 ± 1	0.03
Hypertension	42.7%	60.6%	0.01
Diabetes Mellitus	5.2%	6.2%	0.77
HDL-cholesterol, mmol/l	1.24 [1.01–1.56]	1.18 [0.93–1.47]	0.29
Total cholesterol, mmol/l	5.9 ± 0.10	5.7 ± 0.11	0.14
LDL cholesterol, mmol/l	4.0 ± 1.0	3.8 ± 1.0	0.15
Triglycerides, mmol/l	1.2 [0.9–1.9]	1.3 [0.9–1.7]	0.49
Creatinine (μmol/L)	84 [75–95]	86 [78–105]	0.07
Creatinine clearance (mL/min/1.73 m^2^)	90.9 ± 25.7	81.2 ± 23.4	0.01
Moderate renal dysfunction	14.0%	40.2%	<0.01
C-reactive protein (mg/L)	1.55 [0.66–2.81]	2.28 [1.18–4.81]	<0.01
Urinary albumin excretion	11.95 [7.07–21.44]	17.75 [8.92–45.35]	<0.01
Alcohol intake > = 1 drink per day	24.7 %	23.7 %	0.87

Except for the CETP I405V genotype (p = 0.03), all polymorphisms examined in this study were in HWE both in the cases as in the controls. The frequencies of the genotypes are listed in Table 2. In the same table, the results of the association study using the dominant and recessive models are given. The polymorphisms of the RAS and Bradykinin gene did no show a significant association with AF in this case-control study. The two genetic variations in the CETP gene were associated with AF. The I405V genotype was associated with AF in the recessive model (p = 0.015)and TaqIB in the dominant model (p = 0.005). The Armitage test (not assuming HWE) for trend was used to replicate the associations found using logistic regression. Using the Armitage test, CETP I405V was not significantly associated with AF (p for trend = 0.34). CETP TaqIB polymorphism remained significant associated with AF (p for trend = 0.03). After adjustment for other significant risk factors predisposing to AF (presence of electrocardiographic LVH, ischemic heart disease, hypertension, renal dysfunction, elevated CRP, and micro- or macroalbuminuria), the association between CETP TaqIB polymorphism and AF remained significant (Odds ratio [95% confidence intervals] for AF: 0.35 [0.16–0.76], p = 0.008).

**Table 2:** Distribution of genotypes in controls without atrial fibrillation (AF) and subjects with AF.

**Locus**	**AF Controls (n = 97)**	**AF Cases (n = 97)**	P value (Chi-square) OR (95%CI)*
ACE gene I/D			0.44
II	22.7%	31.1%	0.65 (0.33–1.27)
ID	50.0%	43.3%	0.92 (0.47–1.78)
DD	27.3%	25.6%	
AGT gene G-6A			0.96
AA	16.1%	17.6%	1.07 (0.59–1.97)
AG	46.0%	46.2%	1.11 (0.51–2.44)
GG	37.9%	36.3%	
AT1R gene A1166C			0.28
CC	8.0%	11.6%	0.73 (0.41–1.32)
AC	46.0%	34.7%	1.50 (0.55–4.05)
AA	46.0%	53.7%	
Bradykinin 2 C58T CC	34.1%	36.8%	0.92 0.89 (0.48–1.63)
TT	18.2%	16.8%	0.91 (0.43–1.96)
CT	47.7%	46.3%	
Bradykinin 2 C181T			0.54
CC	72.7%	76.3%	0.83 (0.42–1.62)
TT	1.1%		n.a. (empty cells)
CT	26.1%	23.7%	
Bradykinin 2 exon1			0.35
-9/-9	38.2%	29.3%	1.08 (0.60–1.94)
-9/+9	43.8%	45.7%	1.49 (0.80–2.77)
+9/+9	18.0%	25.0%	1.52 (0.74–3.12)
CETP I405V			0.02
AA	44.3%	46.8%	0.90 (0.50–1.62)
AG	52.3%	38.3%	4.96 (1.37–17.90)
GG	3.4%	14.9%	
CETP TaqIB			0.02
B1B1	24.1%	44.1%	0.40 (0.21–0.77)
B1B2	62.1%	43.0%	0.93 (0.39–2.19)
B2B2	13.8%	12.9%	

* Odds ratios (95% Confidence Intervals) for the presence of AF on the electrocardiogram were obtained by logistic regression; the top OR is for autosomal dominant model and the bottom for autosomal recessive model.

**Figure 1:**
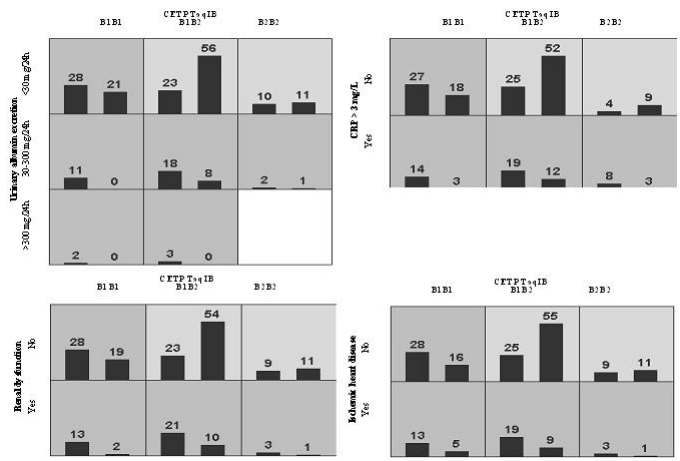
**Distribution of high-risk (dark shading) and low-risk (light shading) combinations associated with atrial fibrillation in the four significant gene-environment models using Multifactor Dimensionality Reduction (MDR) analysis. **The percentage of patients with atrial fibrillation (left bar in boxes) and matched control subjects (right bar in boxes) is shown for each combination. Boxes were labeled as high-risk (dark-shaded) if the ratio of the percentage of cases to controls met or exceeded the threshold of 1.0.

Table 3 and Figure 1 shows the best interaction models to explain AF in this study as determined by MDR analysis. The overall best interaction model included the combination of micro- or macroalbuminuria and CETP TaqIB polymorphism. Other significant models were the combination of CRP >3 mg/L and the CETP TaqIB polymorphism, the combination of renal dysfunction and the CETP TaqIB polymorphism, and the combination of ischemic heart disease and CETP TaqIB polymorphism (P < 0.05). Similar significant interactions were found for the CETP I405V polymorphism (data not shown because of deviation HWE).

**Table 3:** Summary of Multi-Factor Dimensionality (MDR) results

**Model**	**Training Accuracy**	**Testing Accuracy**	P-value*	**CVC†**
CETP TaqIB – Micro or Macroalbuminuria	0.6761	0.6283	0.04	8/10
CETP TaqIB – C-reactive protein>3 mg/L	0.6650	0.6661	0.002	NA
CETP TaqIB – Renal dysfunction	0.6701	0.6728	0.002	NA
CETP TaqIB – Ischemic heart disease	0.6650	0.6578	0.006	NA

* 1000 fold permutation test

**† **Cross-validation Consistency

**Figure 2:**
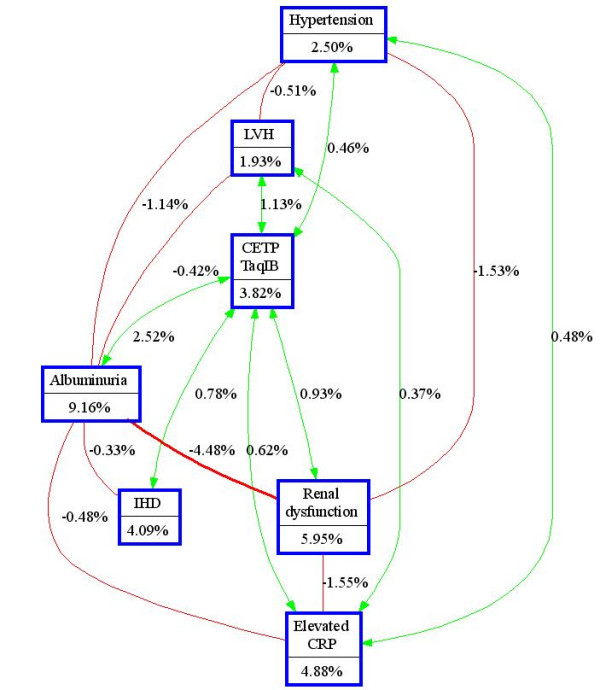
**Interaction entropy graph. **The interaction models illustrated in the interaction graph describe the percent of the entropy in case-control status (i.e. presence of atrial fibrillation) that is explained by each factor or two way interaction. Each gene or environmental factor is shown in a box with the percent of entropy below the label. Two way interactions between factors are depicted as an arrow accompanied by a percent of entropy explained by that interaction. Redundancy is depicted as a line between factors accompanied by a negative percent of entropy. LVH: Left ventricular hypertrophy. IHD: Ischemic heart disease. CRP: C-reactive protein.

After identifying the high-risk combinations of factors using MDR, we applied interaction entropy algorithms to visualize the observed interactions and to determine whether they are additive or non-additive (Figure 2). Entropy analysis showed a small synergistic interaction between the presence of albuminuria and CETP TaqIB polymorphism on AF. Next to the independent effects of both factors, the combination removes an additional 2.52% of the total entropy. The other three significant interactions found by MDR are mostly additive as illustrated by the interaction graph.

## Discussion

This study showed for the first time an association between the CETP TaqIB polymorphism and AF. Using the MDR method, we established that the CETP TaqIB polymorphism in association with other risk factors of AF, i.e. albuminuria, elevated CRP, renal dysfunction, and presence of ischemic heart disease could predict AF even more strongly. Other genetic polymorphisms of genes encoding bradykinin and elements of the RAS did not correlate with the presence of AF.

The underlying biological mechanism explaining the association between AF and the CETP TaqIB polymorphism remains unclear in this study. A logical explanation would be that the widely acknowledged association between CETP gene variations (resulting in lower plasma CETP) and higher HDL cholesterol levels. [12] may prevent the initiation of (preclinical) atherosclerosis and subsequently the development of AF. This mechanism is very likely considering the modulating effect of ischemic heart disease on the association between CETP TaqIB polymorphism and AF in the present study.

Data corroborating this have recently been published. Some studies have linked CETP polymorphisms to cardiovascular disease, hypertension, and even longevity. [13,31]. Since new-onset AF in about 40% of the cases is linked to either coronary artery disease, hypertension, or both. [32], genetic variants impacting the CETP mass may provide a pathophysiologically rational correlate of AF. However, ancillary mechanisms such as inflammation, oxidative stress and use of alcohol cannot be excluded as the link between CETP and the predisposition to AF. Decreased CETP activity in the subjects who have the B2B2 genotype of the CETP TaqIB polymorphism might prevent oxidation of LDL and decrease inflammation of the vascular wall through its effect on HDL [11]. In this study, subjects with AF had lower levels of HDL cholesterol, but this difference did not reach statistical significance due to the small population.

It has recently been recognized that oxidative stress and inflammation may facilitate the development of AF [33]. The fact that the combination of an elevated CRP and CETP TaqIB polymorphism was more powerful in predicting AF than either one alone indicate a potential role of inflammation as underlying mechanism. Another common pathway might be the presence of insulin resistance, which has been suggested to contribute to the development of AF [34]. Variations in the CETP gene might influence the different components of insulin resistance [35].

The significant interaction between CETP TaqIB polymorphism and renal dysfunction and especially the presence of albuminuria in predicting AF, pleads for (at least in part) accessory pathway involving the renin-angiotensin system. One common denominator could be the altered expression of liver X receptor, which in turn might modulate CETP expression as well as renin expression, although this is highly speculative. [36,37].

The MDR method indicated four significant two-way interaction models, but did not specify whether there is a synergistic relationship. Therefore, we created an interaction entropy graph to visualize and interpret the observed interactions. This graph showed that the interaction between albuminuria and CETP TaqIB was (partly) synergistic in the presence of significant main effects. If the interaction between albuminuria and CETP TaqIB was purely additive, the total amount of entropy removed would be 13.0%, but instead the total amount of entropy removed by the combination of CETP TaqIB and albuminuria was 15.5%, indicating a synergistic interaction. On the other hand, an additive interaction was observed between the CETP TaqIB polymorphism and elevated C-reactive protein, presence of renal dysfunction or ischemic heart disease. It is therefore more likely that the found interactions between the CETP TaqIB polymorphism and elevated C-reactive protein, presence of renal dysfunction or ischemic heart disease are not biological meaningful.

The relation between polymorphisms of the renin-angiotensin system and AF found by Tsai et al [7]. could not be replicated in our case-control study. Possibly, our study was underpowered. It is important to note the difference in ethnicity between the two samples (Caucasian vs. Taiwanese). It is possible that the RAS genes influence susceptibility to AF differently in the context of different genetic backgrounds. Moreover, studies of genetic polymorphisms of the RAS have been generating conflicting results, possibly due to small sample size, inadequate study populations, and unconditional statistics. A contributory role of genetic variants of the ACE, angiotensin, and AT1R genes in AF is in our opinion still open to debate.

A limitation of our study is the relatively small sample size. The CETP I405V polymorphism deviated from HWE in the control group which could arise due to the small sample of the population and the results of this CETP polymorphism needs to be interpreted with caution. On the other hand, the MDR method, which has been validated in studies with small samples, confirmed the association between CETP TaqIB polymorphism and AF detected by traditional logistic regression analyses. Furthermore, the Armitage test for trend in proportions which does not assume HWE, showed a significant trend between the CETP TaqIB polymorphism and AF. However, the CETP TaqIB polymorphism is not likely to be functional and is probably a marker for one or several functional variants located within the CETP gene or its vicinity. Thus, the results found in this study need to be confirmed in another population preferable with a larger size. Until then, these data are rather hypothesis generating than definite.

Additional studies need also to be done to establish the underlying mechanisms explaining the association between CETP TaqIB polymorphisms and AF and to understand the interactions among CETP TaqIB polymorphism and presence of micro- or macroalbuminuria, elevated CRP, renal dysfunction, and ischemic heart disease. Clinical trials investigating pharmacological inhibitors of CETP might consider to include AF as exploratory outcome.

Also, in this population based cohort, no clinical information such as echocardiographic features could be obtained due to the epidemiological nature of the study. In addition, the type of AF (paroxysmal or persistent) could not be determined, because one standard 12 lead electrocardiogram was made during two minutes.

## Conclusion

In summary, we observed an association between AF and TaqIB polymorphism of the CETP gene. Using the MDR method, we showed that the combination of CETP TaqIB polymorphism and the presence of albuminuria, an elevated CRP, renal dysfunction or ischemic heart disease are the best models for predicting AF in this nested case-control study. Further studies are warranted to further elucidate this association and to investigate whether a true biological link exits between CETP and AF or that both are associated with a common factor such as e.g. atherosclerosis, hypertension, inflammation, oxidative stress or alcohol intake.

## Competing interests

The author(s) declare that they have no competing interests.

## Authors' contributions

FA was involved in the design of the study, performed the statistical analyses and drafted the first and final version of the manuscript. JM was involved in the MDR analyses, creating the interaction entropy graph, and revising the manuscript critically for important intellectual content. MvdB was involved in the design of the study and revising the manuscript critically for important intellectual content. ER was involved in revising the manuscript critically for important intellectual content. RdB was involved in revising the manuscript critically for important intellectual content. RD critically revised the manuscript for important intellectual content. GN critically revised the manuscript for important intellectual content. WvG participated in the design of the study and revising the manuscript critically for important intellectual content.

## Pre-publication history

The pre-publication history for this paper can be accessed here:


